# Good functional outcomes and radiological union following arthroscopic knotless anchor fixation of tibial spine avulsion fractures: A prospective clinical study

**DOI:** 10.1002/jeo2.70692

**Published:** 2026-03-19

**Authors:** Mukesh Laddha, Lakshya Bhardwaj, Luca Farinelli, Amit Meena

**Affiliations:** ^1^ RNH Hospital Nagpur India; ^2^ JMCH Jodhpur Rajasthan India; ^3^ Department of Clinical and Molecular Sciences Clinical Orthopaedics Ancona Italy; ^4^ IRCCS INRCA Ancona Italy; ^5^ KNEECARES – the superspeciality knee clinic Jaipur India; ^6^ Division of Orthopaedic Surgery Queen's University Kingston Ontario Canada

**Keywords:** ARIF, displaced tibial fracture, tibial eminence

## Abstract

**Purpose:**

Tibial spine avulsion fractures compromise ACL function and are common in children and adolescents. Traditional fixation methods may risk physeal injury and intra‐articular damage. Arthroscopic suture‐based techniques offer a minimally invasive, physeal‐sparing alternative.

**Methods:**

Twenty‐five patients (mean age: 19 years) with type II–IV tibial spine fractures underwent arthroscopic anterior row suture fixation using knotless anchors. Functional outcomes as Lysholm, Internation Knee Documentation Committee rating score (IKDC), Range of motion (ROM), stability, and radiological healing were assessed over 24 months.

**Results:**

All patients achieved radiographic union (mean: 16 weeks). Final follow‐up showed restoration of pre‐injury Lysholm (94 ± 5 to 93.6 ± 0.8), IKDC (84 ± 7 to 83.9 ± 0.7), and ROM (142°–140°). No instability, growth disturbance, or hardware‐related complications were observed.

**Conclusion:**

This technique provides stable fixation, preserves the physis, and avoids intra‐articular hardware. Functional and radiological outcomes were excellent, supporting arthroscopic suture fixation as a safe and effective option for tibial spine avulsion fractures.

**Level of Evidence:**

Level III, prospective study.

## Graphical abstract

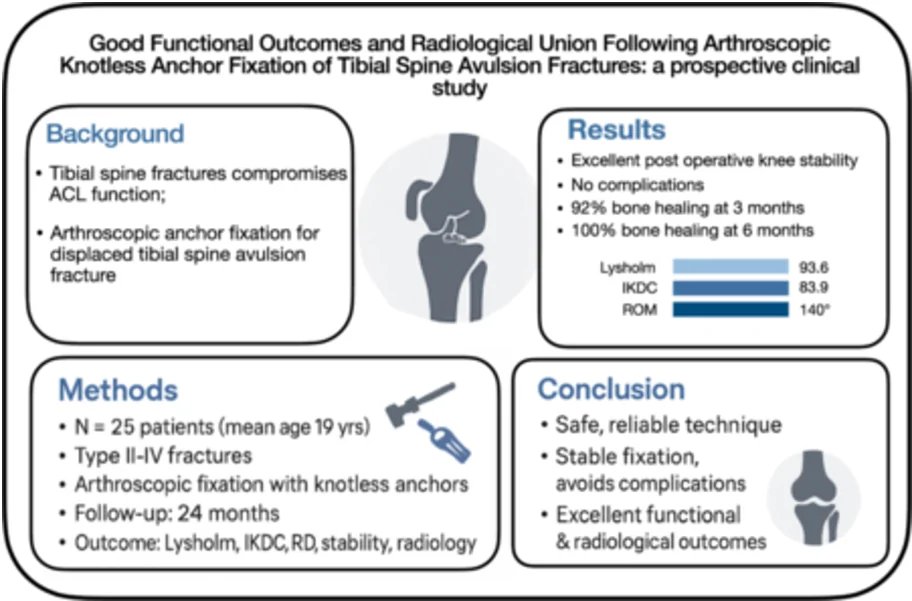

**Abbreviations**
ACL: anterior cruciate ligament
AL: anterolateral
AM: anteromedial
IKDC: International Knee Documentation Committee
MCID: minimal clinically important difference
PCL: posterior cruciate ligament
ROM: range of motion

## INTRODUCTION

Tibial spine avulsion fractures result in anterior cruciate ligament (ACL) dysfunction and are predominantly observed in children aged 8–14 years [16]. The injury mechanism typically involves hyperextension, rotational forces, or abrupt deceleration during athletic activities. In adults, these fractures are more commonly associated with high‐energy trauma, such as motor vehicle accidents [25].

In the paediatric population, the epiphyseal bone demonstrates reduced resistance to traction forces compared to the ACL substance, rendering it more susceptible to avulsion injuries [38]. ACL avulsion fractures are classified into non‐displaced (Type I), partially displaced or hinged (Type II), and completely displaced (Type III) categories. Type III fractures are further subdivided into ‘non‐rotated’ and ‘rotated’ variants [26], with a subsequent modification introducing comminuted avulsion fractures (Type IV) [41]. These injuries may lead to knee instability, and the presence of intra‐articular fragments can result in mechanical obstruction of joint motion [13]. Anatomical reduction and stable internal fixation are essential to restore normal knee biomechanics [16]. Previous studies suggest that undisplaced or minimally displaced fragments may be managed conservatively [28], whereas displaced fragments typically require open or arthroscopic reduction and internal fixation (ORIF/ARIF) [21, 31]. However, current fixation techniques are associated with several complications, including the creation of large bone tunnels that compromise structural integrity, physeal injury in skeletally immature patients due to transphyseal fixation (potentially leading to growth disturbances [1]), damage to intra‐articular structures during tunnel placement, and iatrogenic comminution of the avulsed fragment. Additional complications include residual laxity secondary to plastic deformation of the ACL or inadequate reduction resulting in non‐union [22]. Recent literature increasingly supports arthroscopic fixation as the preferred approach. Emerging techniques—such as mini‐tunnel suture bridges [30], Orthocord suture fixation [42], and physeal‐sparing K‐wire fixation in skeletally immature patients [6] have demonstrated excellent clinical and radiological outcomes. Given these considerations, physeal‐sparing techniques were favoured for their ability to minimise iatrogenic damage to intra‐articular structures. Based on this rationale, a novel arthroscopic technique for tibial spine fixation has been developed.

The present study aims to evaluate patient‐reported functional outcomes (Lysholm knee score and IKDC), range of motion (ROM), fixation stability, and radiological healing. We hypothesise that our technique will yield favourable functional and radiological outcomes.

## METHODS

### Study design and participants

A prospective clinical observational study was conducted after ethical approval from the institute's ethics committee (n.328/22 of RNH Hospital). The inclusion criteria were consecutive patients aged 8–40 years with type II, III or IV tibial spine avulsion fractures who presented acutely after injury from January 2022 to December 2022. Patients were excluded if there was concomitant meniscus or PCL, collateral or femoral cartilage injuries. Patients were also excluded if there were other fractures in the limb. Clinical assessment was done using the Lachman test and pivot shift test to determine laxity; radiological evaluation included plain X‐rays and Magnetic resonance imaging (MRI).

### Surgical technique

Under spinal anaesthesia, the patient is placed in the supine leg‐dangling position. Pneumatic thigh tourniquet inflated. Standard anterolateral (AL) and anteromedial (AM) portals are made. Viewing from the AL portal, the first step is to identify the fracture fragment and free the entrapped meniscus. Through the same viewing portal, the joint and fracture bed is cleared of haematoma, loose chondral or osteochondral fragments (Figure 1a,b). The fracture fragment is reduced with the help of a probe and temporarily fixed with one 2‐mm K‐wire inserted percutaneously (Figure 1c). Then, a self‐retrieving FirstPass Mini device (Smith & Nephew, Andover, MA) preloaded with fibre tape is introduced from the anteromedial portal to take a bite in the substance of the ACL in the centre of its footprint (Figure 1d,e). The next step is to create a low transpatellar portal via an outside‐in technique using an 18‐gauge spinal needle with the scope in the AL portal (Figure 1f). A Passport cannula (Arthrex) is inserted in the transpatellar tendon portal. Then, viewing from the same AL portal, the fibre tape is retrieved from the low transpatellar portal. The fibre tape is loaded onto a 5.5‐mm SwiveLock anchor (Arthrex, Naples, FL) and inserted in the anterior tibia after properly tensioning the fibre tape, which is holding and reducing the fracture fragment (Figure 1g). Finally, after the fibre tape is cut, the knee is taken through a full ROM and reduction is maintained without any anterior beaking (Figure 1h). If the ACL avulsion fragment is larger or comminuted, tapes and two Knotless anchors are placed anterior at a distance of 8–10 mm, giving uniform compression through the fracture fragment and also helping to avoid anterior beaking (Figure 2) [20].

**Figure 1:**
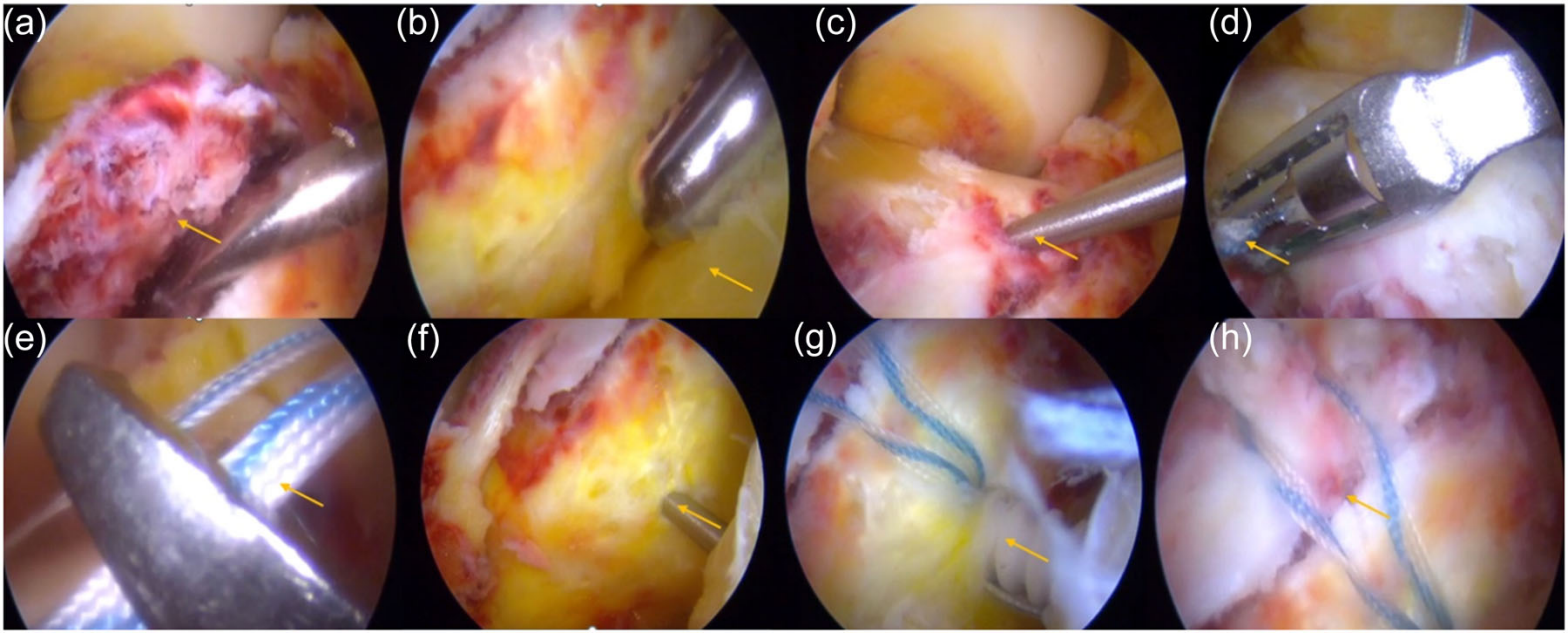
Arthroscopic visuals of anterior row fixation of avulsed tibial spine with a single knotless anchor. (a) Arrow shows an avulsed bone fragment. (b) Arrow shows footprint preparation. (c) Arrow shows temporary fixation with k K‐wire after reduction. (d) Arrow shows bite of fibre tape taken in ACL substance with first pass. (e) Fibre tapes retrieved. (f) Arrow shows pilot hole drilling for knotless anchor insertion at the anterior tibia. (g) Arrow shows a knotless anchor with engaged fibre tape. (h) Arrow shows appropriately reduced tibial sine fragment.

**Figure 2:**
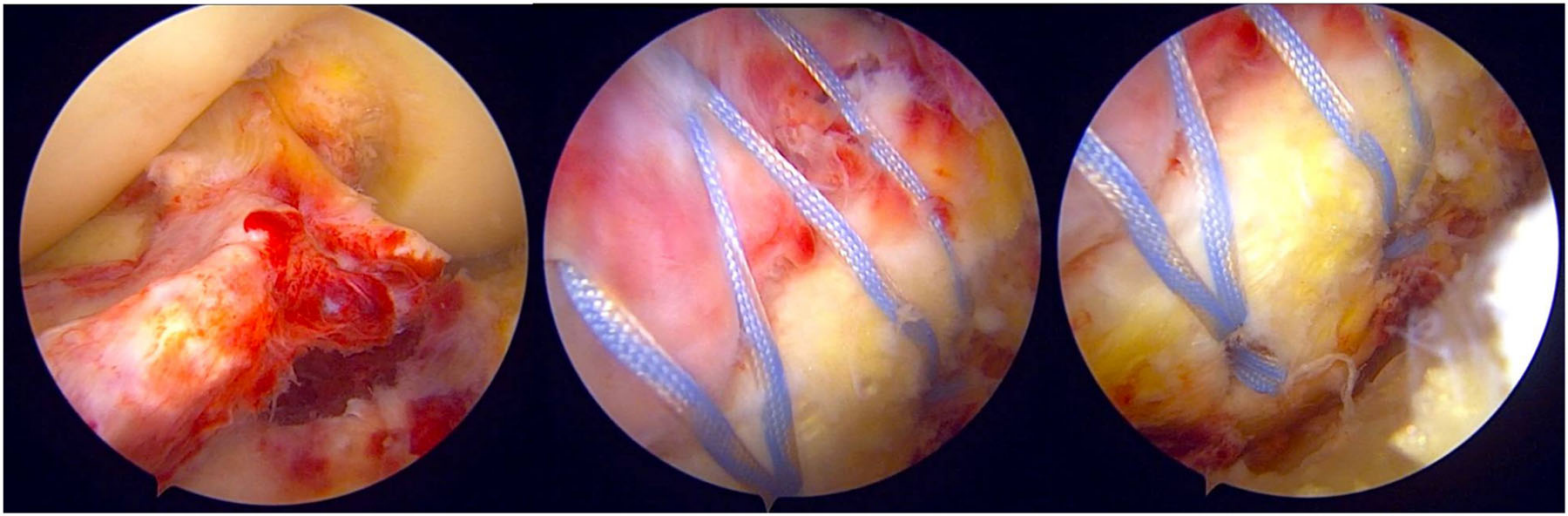
Arthroscopic visuals of anterior row fixation of a larger avulsed tibial spine with two anchors.

### Post operative rehabilitation protocol

Rehabilitation was started on the first day after surgery with patellar mobilisations and quadriceps isometric strengthening exercises. All patients had a locking knee brace in 0° extension for 2 weeks, 0°–60° for 1 week and then increased 10°–20° per week individually according to the patient's compliance. They were allowed full weight‐bearing mobilisation with knee brace 3 weeks after the surgery, the brace was removed after 4 weeks from surgery.

### Follow‐up protocol

Patients were routinely assessed at 30 days, 3 months, 6 months, 12 and 24 months after surgery. At follow‐up, patients were assessed for Lysholm knee score, IKDC and ROM; Lachman and pivot shift test were recorded pre‐operatively under anaesthesia and post‐operatively at follow‐up visits by senior author. As regards to pivot shift test, the International Knee Documentation Committee (IKDC) classification defined the pivot shift test as follows: Grade 0 (normal), Grade 1 (glide), Grade 2 (clunk) or Grade 3 (locked subluxation) [11]. The anterior drawer test was performed by the senior author and was considered positive when anterior tibial translation exceeded 5 mm compared to the contralateral side [9]. Postoperative radiographs were taken at 1, 3 and 6 months after surgery to assess fracture healing. Growth disturbances in skeletally immature patients were monitored through annual clinical evaluations. When clinical findings raised suspicion, standardised radiographic assessment of the pelvis and lower limbs was performed and subsequently reviewed by the senior author.

### Statistical analysis

All statistical analyses were performed using Microsoft Excel software. Continuous variables were presented as mean ± standard deviation (SD). Normality of data distribution was assessed using the Shapiro‐Wilk test. Paired t‐tests were used to compare pre‐operative and post‐operative continuous variables for normally distributed data, while Wilcoxon signed‐rank tests were used for non‐normally distributed data. The minimal clinically important difference (MCID) was defined according to previous papers about ligament surgery: 10 points for Lysholm and 14 points for IKDC. These thresholds were used to interpret the clinical relevance of changes over time [27]. Categorical variables, such as the presence or absence of instability (Lachman test, Anterior drawer test), were analysed using the Chi‐square test or Fisher's exact test, as appropriate. A *p*‐value < 0.05 was considered statistically significant.

## RESULTS

### Study population

A total of consecutive 25 patients with tibial spine avulsion fractures were included in the study. There were 15 males and 10 females, with a mean age of 19 years (range: 11–37 years). In the present study, the median time (Interquartile range, 1st–3rd quartile) from injury to surgery was 7 (3–15) days. Therefore, the patient‐reported pre‐injury scores were recorded and used as a baseline rather than a pre‐operative score. Patients' characteristics were summarised in (Table 1). No patients have been lost to follow‐up.

**Table 1:** Patient's characteristics.

Variables	Screw fixation (*N* = 25)
Age, mean (SD) [range]	19 (4.5) [11–37]
Gender	
Male, *n* (%)	15 (60%)
Female, *n* (%)	10 (40%)
Meyers and McKeever classification, *n* (%)	
II	7 (28%)
III	15 (60%)
IV	3 (12%)
Time from injury to surgery, days (SD) [range]	7 (2.8) [3–15]
Open physis	7 (28%)
Final follow‐up	24 months
Complications	0

### Patients reported outcomes measures and ROM

In the present prospective study all three primary outcomes—ROM, Lysholm score, and IKDC score—showed early postoperative decline followed by progressive recovery (Table 2). ROM decreased from a pre‐injury mean of 142° (0.8) to 130.2° (4.6) at 3 months, then improved to 133° (4.4) at 6 months, 137° (6.3) at 12 months, and 140° (2.4) at 24 months. Lysholm scores dropped from 94 (5.0) pre‐injury to 89.1 (5.6) at 3 months, returning to 93 (1.0) at 12 months and 93.6 (0.8) at 24 months. IKDC scores followed a similar trend with 84 (7) pre‐injury; 81.6 (3.3) at 3 months and 83.9 (0.7) at 24 months. Wilcoxon signed‐rank tests revealed no statistically significant differences across time points (all *p*  >  0.05). Importantly, changes never exceeded the minimal clinically important difference (MCID) thresholds of 10 points for Lysholm and 14 points for IKDC, indicating that functional recovery was maintained throughout follow‐up. Stability tests (Lachman and Pivot Shift Test) were negative in all patients at final follow‐up. Radiographic union was achieved in 23 (92%) patients at 3 months post operatively, whereas 2 patients (8%) showed complete radiographic union at 6 months after surgery (Figures 3 and 4). No case of non‐union, malunion, or growth disturbance was observed in patients with open physes. There were no incision‐related complications, infections, or neurovascular injuries, hardware‐related symptoms, or re‐operation reported.

**Table 2:** ROM, Lysholm score and IKDC at different follow‐up.

Variables	Pre‐injury	3 m	6 m	12 m	24 m	*p*‐Value
ROM, mean (SD)	142 (0.8)	130.2 (4.6)	133 (4.4)	137 (6.3)	140 (2.4)	n.s
Lysholm score, mean (SD)	94 (5.0)	89.1 (5.6)	92.4 (2.8)	93 (1.0)	93.6 (0.8)	n.s.
IKDC score, mean (SD)	84 (7)	81.6 (3.3)	82.9 (1.7)	83.5 (0.9)	83.9 (0.7)	n.s

Abbreviations: IKDC, International Knee Documentation Committee; m, months; n.s., not significant; ROM, range of motion; SD, standard deviation.

**Figure 3:**
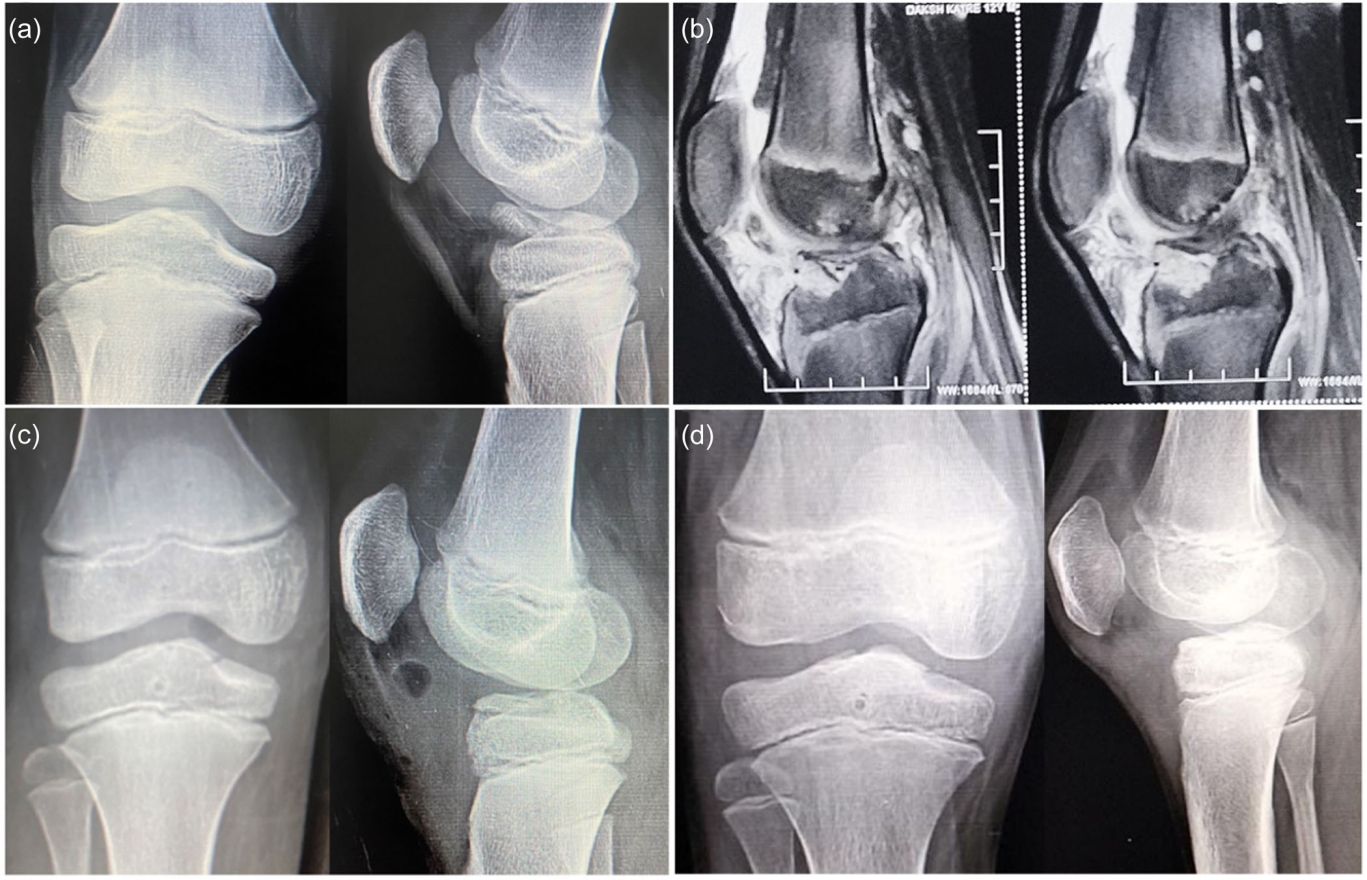
(a) Preoperative X‐ray suggestive of avulsed tibial spine. (b) Preoperative MRI suggestive of avulsed tibial spine. (c) Postoperative X‐ray suggestive of well‐reduced fractured fragment with physeal‐sparing fixation. (d) 3‐month follow‐up X‐ray suggestive of complete radiological union. MRI, magnetic resonance imaging.

**Figure 4:**
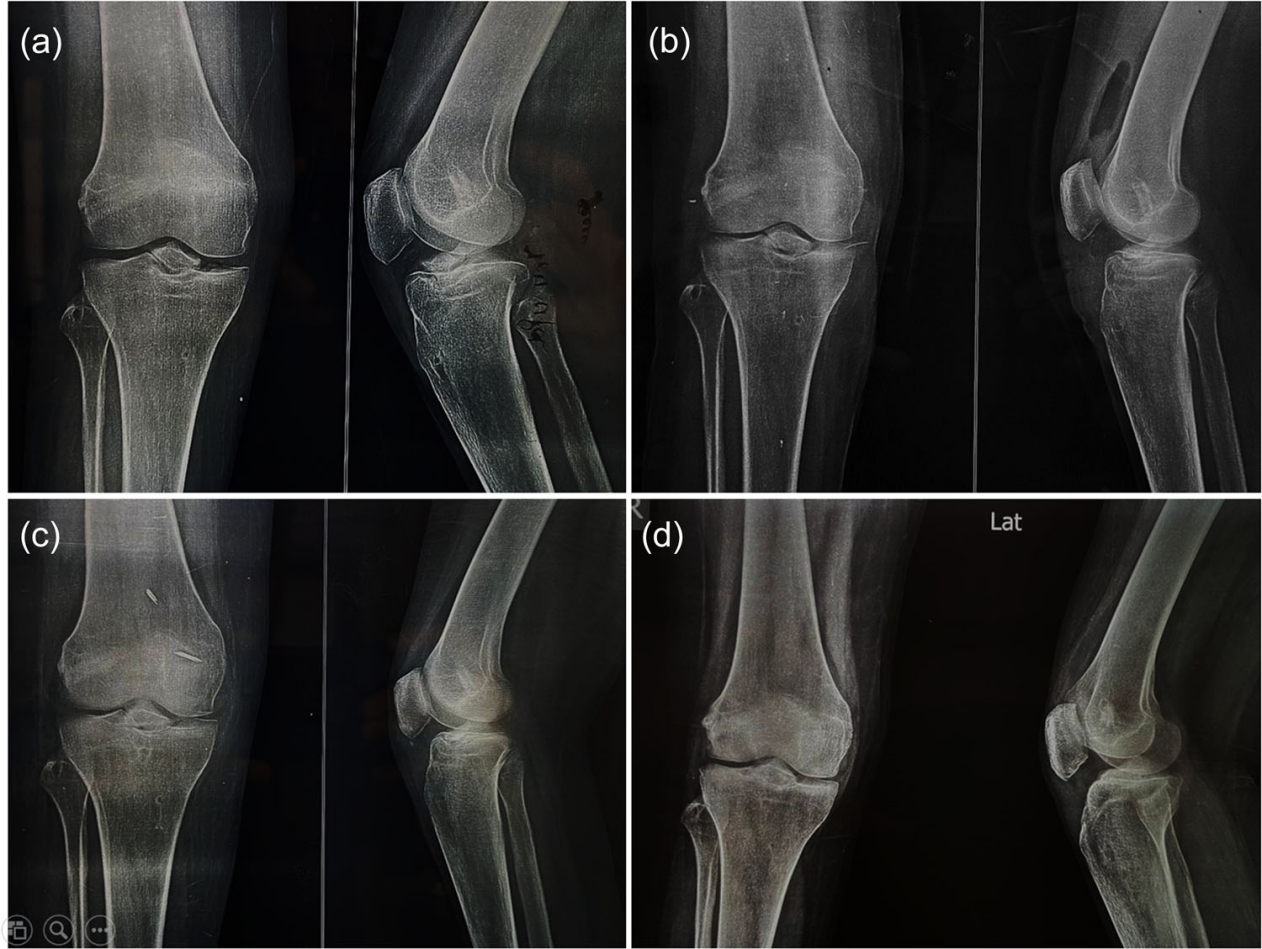
(a) Preoperative X‐ray suggestive of non‐united avulsed tibial spine. (b) Postoperative X‐ray suggestive of a well‐reduced fractured fragment with freshened fractured margins. (c) 3‐month follow‐up X‐ray suggestive of uniting fracture fragment. (d) 6‐month follow‐up X‐ray suggestive of complete radiological union.

## DISCUSSION

The present study demonstrates that arthroscopic suture fixation of anterior tibial spine (ACL avulsion) fractures offers a stable, safe, and reproducible method of fixation while preserving the physis and eliminating intra‐articular iatrogenic injuries. This all‐inside construct achieved anatomic reduction with excellent functional recovery mean IKDC and Lysholm scores of 83.9 and 93.6, respectively—comparable with or superior to previously reported series. Ahn et al. reported Lysholm scores of 95.6 using suture fixation [2], Song et al. 89.5 with suture‐anchor constructs [35], and Jang et al. 98 with a three‐point suture technique [15]. These findings align with contemporary studies advocating arthroscopic, minimally invasive, and physeal‐sparing approaches that provide robust fixation strength and favourable outcomes [4, 5, 30].

A major advantage of this technique is the absence of intra‐articular screws or metallic implants, thereby avoiding complications such as fragment comminution, implant migration, or impingement that occasionally occur with screw fixation [33]. Importantly, our method safeguards the open tibial physis, minimising the risk of growth‐plate disturbance or hyperextension deformity, which is a key concern in skeletally immature patients [6] Bauer et al. [5] and Pu et al. [30] described all‐suture and mini‐tunnel bridge constructs that achieved secure fixation without crossing the physis, while Du et al. [8] and Chu et al. [7] confirmed excellent outcomes with modified suture‐bridge and double‐row anchor techniques. Collectively, these findings reinforce that growth‐plate preservation and anatomic reduction remain cornerstones of modern management of tibial spine fractures.

Functionally, the present results parallel those of other contemporary cohorts. Yu et al. [39] and Yuan et al. [40] achieved Lysholm scores exceeding 95 using anchor‐based arthroscopic fixation, comparable to our outcomes. Paediatric and adolescent studies by Jackson et al. [14] and Uboldi et al. [37] reported similar functional recovery and early return to activity, highlighting that suture fixation ensures appropriate ligament tension restoration even in skeletally immature knees.

Biomechanical evidence supports these clinical observations. Experimental models indicate that stable fixation should resist cyclic loads of 300–450 N to prevent displacement [38]. Anderson et al. [3] and Eggers et al. [10] demonstrated that suture constructs possess superior resistance to cyclic loading and pull‐out forces compared with screws. Recent studies have validated these principles using re‐tensionable all‐suture systems capable of maintaining stability under physiological conditions [36]. These constructs provide strong fixation while simplifying any future ACL reconstruction because they avoid metallic hardware that might obstruct tunnel placement.

Historically, fixation strategies evolved from open reduction using pins, K‐wires, or screws [17, 22, 29] toward arthroscopic suture‐based methods that maximise strength and minimise morbidity. Early reports by Meyers and McKeever [25] and later by Zaricznyj [41] established the foundation for modern techniques but were limited by hardware complications and postoperative stiffness. Comparative analyses confirm that screw fixation, while effective, may lead to fragment breakage or necessitate implant removal [32]. In contrast, suture‐based constructs offer equivalent or greater biomechanical stability without intra‐articular hardware. [23]. Recent randomised and retrospective series further support this shift: Badran et al. [4] demonstrated superior outcomes with arthroscopic sutures over open screw fixation, while Zhang et al. [42] found improved union and reduced complications using orthocord suture fixation in 2025.

Postoperative stiffness and arthrofibrosis have historically represented the most frequent complications following tibial spine fixation [24]. Vander Have et al. [12] reported stiffness rates up to 20% after open fixation, often linked to delayed rehabilitation. However, recent protocols emphasise early physiotherapy following stable arthroscopic fixation to prevent soft‐tissue adhesion [34]. Our series recorded no incidence of arthrofibrosis, echoing the experience of Kuang et al. [19], who demonstrated that early mobilisation within 2–4 weeks markedly reduces postoperative stiffness. Stable fixation, early motion, and physeal preservation together form the triad of successful management for tibial spine avulsion injuries.

The present findings strengthen the growing consensus that all‐inside arthroscopic suture fixation represents an optimal balance between mechanical stability and biological safety. It allows anatomic reduction under direct visualisation, preserves growth potential, and avoids secondary surgery for implant removal. Moreover, its reproducibility with standard arthroscopic instruments enhances its utility across varying surgical setups. Although fixation with screws [22], sutures [23], K‐wires [17], or staples [18] has yielded generally favourable results, outcomes have varied due to technical complexity and implant‐related issues. The current suture‐only, physeal‐sparing approach minimises these concerns while ensuring reliable union and excellent function. The continued refinement of suture configurations and multicentric studies comparing biomechanical properties across fixation techniques will further consolidate its role as the preferred surgical option for both adolescent and adult patients.

Limitations of the present study include the modest sample size and lack of a comparative control group. Growth disturbance was assessed only with long‐standing radiographs and not with computer tomography (CT). Objective postoperative laxity assessment (such as KT‐1000 arthrometer testing) and long‐term follow‐up could further validate the durability of this technique. Nonetheless, the consistent achievement of radiological union, absence of instability, and excellent patient‐reported outcomes provide convincing evidence of efficacy.

## CONCLUSIONS

Arthroscopic anterior row suture fixation with knotless anchors provides stable fixation, allows early mobilisation with full functional recovery, avoids hardware‐related complications, and preserves the physis in skeletally immature patients.

## AUTHOR CONTRIBUTIONS

Mukesh Laddha contributed to the conceptualisation of the study, the overall design, data curation, and initial drafting of the manuscript. Lakshya Bhardwaj was responsible for the development of the methodology, statistical analyses, and interpretation of the results, providing substantial critical revisions to the text. Luca Farinelli supervised the clinical aspects of the project, contributed to data interpretation, and was involved in the review, editing, and validation of the final manuscript. Amit Meena coordinated project administration and data acquisition, contributed to visualisation of findings, and provided final approval of the manuscript in its definitive form.

## CONFLICT OF INTEREST STATEMENT

The authors declare no conflicts of interest.

## ETHICS STATEMENT

n.328/22 of RNH Hospital. Informed consent was obtained from all individual participants included in the study.

## Data Availability

The data that supports the findings of this study are available in the supplementary material of this article.
